# Management of High Cardiovascular Risk in Diabetic Patients: Focus on Low Density Lipoprotein Cholesterol and Appropriate Drug Use in General Practice

**DOI:** 10.3389/fcvm.2021.749686

**Published:** 2021-10-25

**Authors:** Michelangelo Rottura, Giulia Scondotto, Maria Antonietta Barbieri, Emanuela Elisa Sorbara, Chiara Nasso, Sebastiano Marino, Riccardo Scoglio, Giuseppe Mandraffino, Giovanni Pallio, Natasha Irrera, Egidio Imbalzano, Giovanni Squadrito, Francesco Squadrito, Vincenzo Arcoraci

**Affiliations:** ^1^Department of Clinical and Experimental Medicine, University of Messina, Messina, Italy; ^2^Italian Society of General Practice, Section Messina, Messina, Italy; ^3^Internal Medicine Unit, Lipid Center, Messina, Italy

**Keywords:** cardiovascular risk, diabetes, lipid-lowering drugs, clinical practice, appropriateness

## Abstract

This study aimed to evaluate the management of high cardiovascular risk (CVr) in the patients with diabetes by exploring the prescribing behavior in a setting of general practitioners (GPs). A retrospective cohort study was carried out using the data recorded between 2018 and 2020 in the clinical database of 10 GPs. Diabetes was defined using the International Classification of Diseases (ICD-9-CM) coding (250^*^) or using the laboratory parameters (hyperglycemia condition: ≥126 mg/dL). A cohort was described stratifying by demographic, clinical and therapeutic characteristics, and laboratory tests. Both the CVr and statin prescriptions were evaluated; adherence to statin therapy (medication possession ratio, MPR ≥ 80) was calculated in accordance with the low-density lipoprotein cholesterol (LDL-C) target. The multivariate logistic regression models with adjusted odds ratios (*OR*s) and the corresponding 95% Confidence Intervals (CIs) were calculated to identify the predictors of lipid modifying agents use and achieved target therapy; moreover, glucose-lowering drugs use was evaluated. Out of 13,206 people screened, 1,851 (14.0%) patients were affected by diabetes mellitus (DM), and 1,373 were identified at high/very high CVr. Of them, 1,158 (84.3%) had at least one measurement of LDL-C, and 808 (58.8%) received a prescription with at least one lipid-lowering drug (LLD). The patients at high/very high CVr treated or not treated with LLD, reached the LDL-C target in 24.0 and 10.3%, respectively (*p* < 0.001). Furthermore, 34.6% of patients treated with high intensity LLDs and adherent to therapy showed the LDL-C values below the therapeutic target. Out of 1,373 patients at high/very high CVr, 958 (69.8%) had at least one prescription of glucose-lowering drugs. Of them, 52.0% (*n* = 498) were prescribed not in agreement with the current guidelines. More specifically, 392 patients (40.9%) were treated with metformin only, while the remaining 106 (11.1%) were treated with metformin together with hypoglycemic agents other than glucagon-like peptide-1 receptor agonists (GLP1-RA) or sodium-glucose-transporter 2 (SGLT2) inhibitors. Our results suggest the urgent need to improve the management of patients with diabetes at high and very high CVr in the real life, to reduce the burden of diabetes on the health system.

## Introduction

Diabetes mellitus (DM) is a metabolic disease characterized by the persistent state of hyperglycemia due to increased insulin resistance and/or decreased pancreas beta cells function (1). The prevalence is still increasing (2): 451 million estimated patients are diagnosed with DM whereas 22.9 million are newly diagnosed (3). The growing number of patients with diabetes has a great impact on the public health system since DM represents one of the key risk factors for cardiovascular risk (CVr) (4). Moreover, DM is often associated with dyslipidemia and low-density lipoprotein cholesterol (LDL-C) values are strongly correlated with CVr; therefore, the decrease of LDL-C in the patients with diabetes may significantly reduce the CVr. For this reason, different LDL-C therapeutic targets should be achieved, considering the CVr profile, classified in *very high, high, moderate, and low* (5).

Several drugs are used in the patients with impaired metabolic profile, such as statins, ezetimibe, bile acid sequestrants, fibrates, omega 3, and lipoprotein inhibitors of the subtilisin/Kexin convertase type 9 (PCSK9-i), even if achieving the LDL-C therapeutic targets (<55/70 mg/dL) of patients with high/very high CVr can be reached only using high-intensity treatments. Awareness of the specific effects of advancing age and comorbidities on CV events, indicate the need to manage risk in an individualized manner, empowering management of the patients with diabetes. The previous guidelines for the management of CVr in the patients with diabetes were published in the European Heart Journal in 2013, but the growing number of CV safety trials in the patients with type 2 DM (T2DM) (Harmony, PIONEER) demonstrated that the use of both glucagon-like peptide-1 receptor agonists (GLP1-RA) and sodium-glucose-transporter 2 (SGLT2) inhibitors is strongly recommended in patients with T2DM with prevalent cardiovascular diseases (CVD) or high/very high CVr and treated with metformin. However, the available data on metformin use are controversial: the results of *United Kingdom Prospective Diabetes Study* (UKPDS) suggest a beneficial effect of metformin use in primary prevention of CVD but the described data of more recent studies weaken its role in the CVD (6–9).

The guidelines strongly suggest SGLT2 inhibitor or GLP1-RA treatment for a patient at high/very high CVr, newly treated with glucose-lowering drugs, regardless of the glycemic control. Metformin could be added to achieve hemoglobin glycate (HbA1c) target, and SGLT2 inhibitors or GLP1-RA are also indicated by the guidelines in metformin-treated patients. The appropriate choice of glucose-lowering drugs together with the lipid-lowering drugs (LLDs) in patients with T2DM should be strongly recommended to reduce CV events based on the risk level. The results of a previous study suggest that the gaps in diabetes care were observed in 2005 and persisted in 2017, with a time span of 12 years, and that the number of patients with diabetes achieving the recommended HbA1c target was inadequate (10), as well as for LDL-C (11). Although the current guidelines recommend a multidisciplinary approach, delivery of care is insufficient. Major gaps are evident between the recommended diabetes care, and the care patients are currently receiving, calling for an improvement in quality and system-based approaches.

Our study aimed to assess the management of CVr in the real world, in a setting of high/very high-risk patients with diabetes patients, by investigating the prescribing behavior in general practice.

## Materials and Methods

### Data Sources

A retrospective cohort study was carried out using the computerized clinical medical record of 10 general practitioners (GPs) in Messina's province (Sicily), during 2018–2020, thus including a total of 13,206 patients. The GPs participating in this project agreed to record data during their daily clinical practice, through their dedicated clinical software, and to send complete and anonymous data about their patients to the unique central database. All the GPs received extensive training in the data collection procedure. Data quality checks were routinely performed through the analysis of several parameters, such as missing patient codes, number of daily filled prescriptions, outlier. Any variation within the defined ranges is investigated and back submitted to each participating GP, to receive immediate feedback about the data quality and completeness. The data quality and completeness have been already validated in the previous drug-utilization studies (12, 13).

A database contains information on each GPs patient, aged at least 18 years old, such as age, sex, weight, height, and body mass index (BMI), information on lifestyle (alcohol, smoke), and several data on diagnostic instrumental and laboratory tests, such as fasting plasma glucose (FPG), glycated hemoglobin (HbA1c), dyastolic blood pressure (DBP), systolic blood pressure (SBP), and lipid profile. All the drugs prescribed during the study period were detailed for each patient, as well as all the morbidities recorded since the registration date on the GP list. The Anatomical Therapeutic Chemical (ATC) classification system was used to code information on the drugs. The diagnoses were coded using the International Classification of Diseases, 9th Revision, Clinical Modification (ICD-9-CM).

### Study Population

All the patients with diabetes, as defined using ICD-9-CM coding (250^*^) or using the laboratory parameters (hyperglycemia condition: ≥126 mg/dL), were selected. Among these, all the patients at high/very-high CVr were identified, according to EAS/ESC guidelines definition (as shown in Supplementary Table S1). The patients were followed until death, disenrollment, or end of the study, whichever occurred first. New users of the glucose-lowering drugs were identified as patients with at least one prescription of glucose-lowering drugs during the study period and without any glucose-lowering drugs treatment in the previous year.

A patient encrypted code has been used to maintain the anonymity. The study protocol was approved by the local Ethical Committee of Messina University Hospital (n° prot. N.0010280/2020; Coordinator Centre).

### Data Analysis

A descriptive analysis was performed to compare all the clinical and demographic characteristics of the study population among the patients with diabetes with high/very-high CVr and moderate CVr.

The descriptive statistics were reported as medians, along with interquartile range (IQR), or absolute frequency and percentages, for continuous and categorical variables, respectively.

Since a not normal distribution of some of the numerical variables was shown after applying the Kolmogorov—Smirnov test for normality, a non-parametric approach was adopted. The Mann–Whitney *U* test for independent sample and two-tailed Pearson's chi-squared test were carried out to compare the continuous and categorical variables, respectively.

A cohort of the patients with diabetes was stratified according to CVr. The cohort was described in terms of demographic (sex, age), comorbidities (identified at cohort entry), Charlson Comorbidity Index (CCI), therapeutic characteristics, and laboratory tests. For the patients with diabetes at high and very-high CVr, LDL-C target was estimated. The LLD prescriptions were identified using ATC code (C10AA^*^, C10BA^*^, C10AX09) and grouped as the high intensity LLDs (rosuvastatin >20 mg, atorvastatin >40 mg; any statin plus ezetimibe) or low/moderate intensity LLDs.

Moreover, the treatments carried out using rosuvastatin >20 mg or atorvastatin >40 mg plus ezetimibe were defined as high intensity lipid-lowering strategy. The high intensity lipid-lowering users were identified and stratified by targeting value, as well as the patients treated according to high intensity lipid-lowering strategy. Adherence to the therapy was calculated as medication possession ratio (MPR) with a cut-off of MPR ≥ 80. The MPR was calculated as the proportion of the number of tablets dispensed over the estimated period of LLD treatment. By assuming a single intake per day, the number of pills corresponded to the numbers of days for which the patient had been prescribed with LLD (14). Since the number of prescriptions filled was used as a proxy for beneficiary status, the users with an MPR ≥ 80% was established as a threshold for adherence (15).

The patients were stratified into four groups according to the MPR and high intensity lipid-lowering drugs use. In the patients with diabetes with at least one prescription of glucose-lowering drugs, appropriateness of treatment, in both the new users and prevalent users, were analyzed according to EAS/ESC guidelines (11).

The glucose-lowering drug prescriptions were identified using the ATC code (A10A^*^ and A10B^*^). The prevalence of use was measured for each drug class as the ratio between the number of patients who received at least one prescription of drug and the total number of patients with diabetes. A number of different classes of drugs used were, also, evaluated. The treatments with SGLT2 inhibitors or GLP1-RAs, alone or in combination with other glucose-lowering drugs, were considered appropriate to improve the CVr.

The blood pressure-lowering drugs prescriptions were identified using the ATC code (C02^*^, C03^*^, C07^*^, C08^*^, and C09^*^). A number of different classes of drugs as well as prevalence of use were evaluated.

In the patients with diabetes at high/very-high CVr, the univariate logistic regression models were performed to identify the predictors of lipid modifying agents use and achieved target therapy. All the variables identified as predictors were included in a stepwise multivariate logistic regression model (backward procedure, α = 5%). Moreover, the univariate and multivariate logistic regression models were performed to identify the predictors of inappropriate glucose-lowering drugs use, using the patients with appropriate prescriptions as comparators. The odds ratios (*OR*s) with 95% Confidence Intervals (CIs) were calculated for each covariate of interest in the univariate (crude OR) and multivariate (adjusted OR) regression models. The goodness of fit of the regression model was carried out by the Hosmer–Lemeshow test for adequacy.

A *p*-value <0.05 was considered statistically significant. All the statistical analyses were performed using the SPSS version 23.0 (IBM Corp., SPSS Statistics, Armonk, NY, USA).

## Results

**Figure 1:**
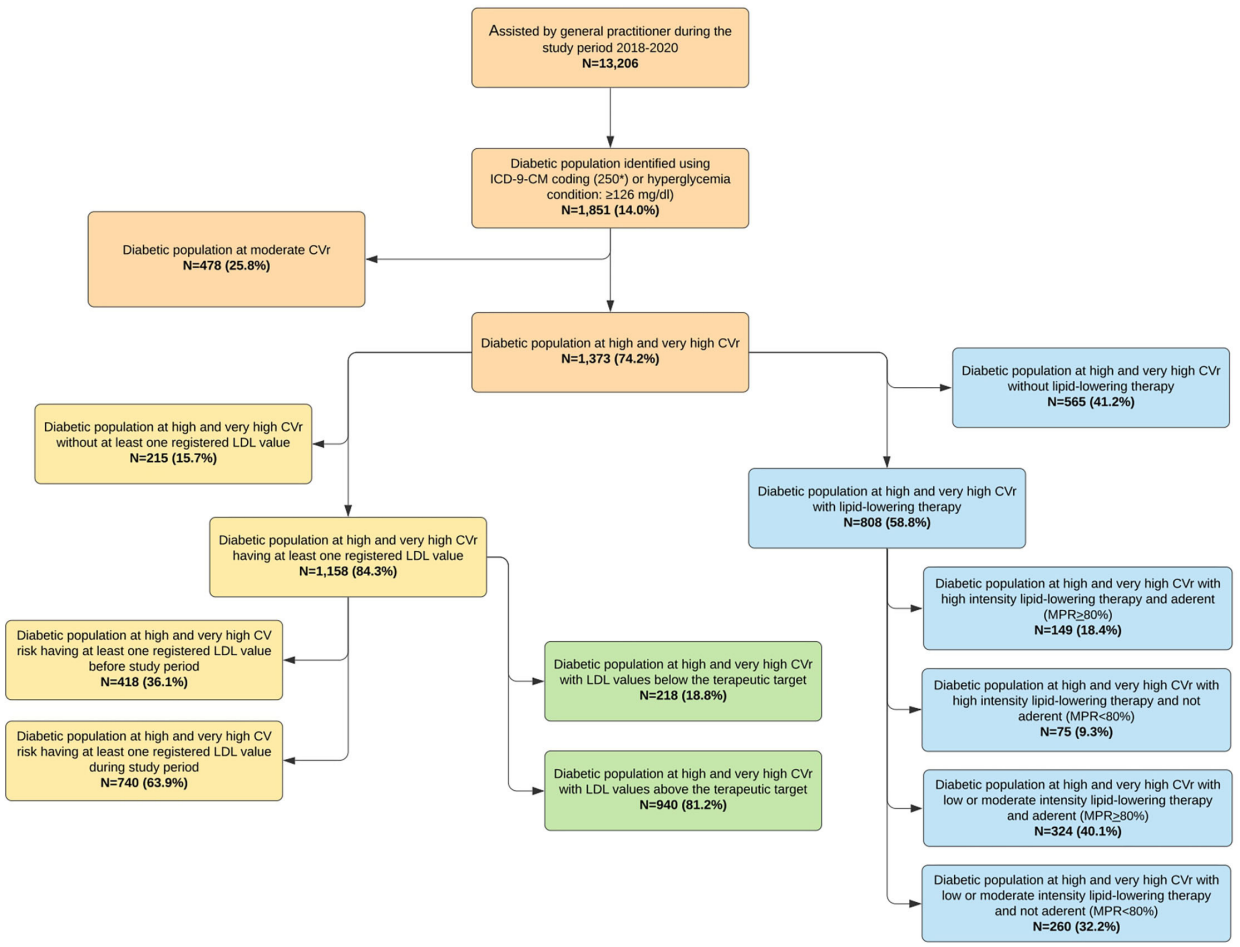
Identification of the study population: focus on the lipidic profile.

A total of 1,851 (14.0%) patients out of 13,206 people covered by the medical care provided by 10 GPs, were diabetic, with a median age (IQR) = 72 (62–80), men = 50.5%. Of these, 1,373 (74.2%) were identified at high or very-high CVr, according to the EAS/ESC guidelines (Figure 1). The patients at high/very-high CVr were significantly older than those with moderate CVr (Table 1), but no significant differences were observed in gender, BMI, total cholesterol, LDL-C, glycemic values, HbA1c, SBP, and DBP between the groups, when they were stratified by CVr. The number of drugs taken throughout the observational period was greater in high/very high CVr patients than in the moderate ones (15, IQR: 9–22 vs. 8, IQR: 3–14; *p* < 0.001). Specialist counseling was carried out in 694 (50.5%) and in 200 (41.8%) patients with diabetes at high/very-high and moderate CVr, respectively (*p* = 0.001). The most frequent comorbidities in the patients with diabetes were hypertension (76.8%) and dyslipidemia (54.8%). Overall, the comorbidities were all more frequent in the patients with high/very-high CVr than in the subjects with moderate CVr, except for neoplasms (Table 1).

**Table 1:** Characteristics of the patient with diabetes stratified by cardiovascular risk.

	**DM patients at high/very high CVr**	**DM patients at moderate CVr**	***P*-value**	**Total**
	***N* = 1,373 (%)**	***N* = 478 (%)**		***N* = 1,851 (%)**
Age (years), median (IQR)	73 (65–81)	65 (55–75)	<0.001	72 (62–80)
Gender, (M)	684 (49.8)	250 (52.3)	0.357	934 (50.5)
BMI	29 (26–33)	29 (26–33)	0.461	29 (26–33)
LDL-C	98 (75–126)	103 (83–125)	0.075	99 (76–125)
Total Cholesterol	175 (147–207)	182 (157–209)	0.085	177 (149–208)
FPG	125 (104–152)	127 (103–148)	0.526	126 (104–151)
HbA1c	6.6 (6.0–7.5)	6.6 (6.1–7.5)	0.265	6.6 (6.1–7.5)
SBP	135 (125–150)	135 (130–146)	0.678	135 (125–150)
DBP	80 (70–85)	80 (70–90)	0.310	80 (70–85)
Comorbidities				
Hypertension	1,217 (88.6)	204 (42.7)	<0.001	1,421 (76.8)
Dyslipidemia	932 (67.9)	82 (17.2)	<0.001	1,014 (54.8)
Arthritis and arthrosis	730 (53.2)	103 (21.5)	<0.001	833 (45.0)
Nephropathy	712 (51.9)	118 (24.7)	<0.001	830 (44.8)
Chronic respiratory diseases	602 (43.8)	100 (20.9)	<0.001	702 (37.9)
Psychic sphere disorders	672 (48.9)	107 (22.4)	<0.001	779 (42.1)
Cerebrovascular disease	594 (43.3)	0.0	NA	594 (32.1)
Osteoporosis	449 (32.7)	62 (13.0)	<0.001	511 (27.6)
Ischemic heart disease	404 (29.4)	0.0	NA	404 (21.8)
Atherosclerosis	357 (26.0)	0.0	NA	357 (19.3)
CKD	251 (18.3)	0.0	NA	251 (13.6)
Neoplasm	204 (14.9)	57 (11.9)	0.113	261 (14.1)
Obesity	223 (16.2)	22 (4.6)	<0.001	245 (13.2)
Gout and metabolism disorders	197 (14.3)	18 (3.8)	<0.001	215 (11.6)
Heart failure	175 (12.7)	8 (1.7)	<0.001	183 (9.9)
CCI	3 (2–5)	1 (1, 2)	<0.001	3 (2–4)

*BMI, Body mass index; CCI, Charlson Comorbidities Index; CKD, chronic kidney disease; CVr, cardiovascular risk; DM, diabetes mellitus; FPG, fasting plasma glucose; IQR, inter quartile range; M, male; LDL-C, low-density lipoprotein cholesterol; HbA1c, glycated hemoglobin; DBP, dyastolic blood pressure; SBP, systolic blood pressure*.

The laboratory tests were recorded more frequently in high/very-high-risk patients with diabetes than in the moderate-risk subjects (LDL-C: 84.3 vs. 63.8%, *p* < 0.001; total cholesterol: 88.1 vs. 68.2%, *p* < 0.001; FPG: 86.5 vs. 69.2%, *p* < 0.001; HbA1c: 74.8 vs. 68.5%, *p* = 0.017, respectively). Furthermore, a similar pattern was observed for BMI, smoking, and alcohol consumption habits in the patients at high/very-high CVr compared with the subjects with moderate CVr (BMI: 51.9 vs. 30.1%, *p* < 0.001; smoke: 34.2 vs. 26, 6%, *p* = 0.002; alcohol abuse: 18.8 vs. 4.0%, *p* < 0.001; respectively).

Al least one glucose-lowering drug was prescribed in 68.2% of patients with diabetes; antihypertensive agents were prescribed in 84.9% of the patients with diabetes and hypertension, while at least one LLD was prescribed in 67.0% of the patients with dyslipidemia. The prevalence of use of different antihypertensive and glucose-lowering drug classes, as well as the number of drug classes per patient are described in the Supplementary Tables S2, S3, S4, and S5.

### Focus on LDL-C and Lipid-Lowering Drugs Use

Among the 1,373 patients with DM at high/very-high CVr, 1,158 (84.3%) had at least one measurement of laboratory test of LDL-C any time, and a measurement was carried out during the 2 years of the study period in 740 (53.9%). Therefore, 633 (46.1%) patients with DM at high/very-high CVr never (or at least in the last 2 years) underwent the assessment of LDL-C laboratory test (Figure 1).

A total of 808 (58.8%) patients at high/very-high CVr received a prescription with at least one LLD; however, no LDL-C value was recorded in 87 (10.8%) subjects.

No treatment or any LDL-C evaluation was performed in 128 patients (9.3%); moreover, 392 (89.7%) out of the remaining 437 patients without the LLD prescriptions during the study period resulted out of LDL-C target.

Within the 808 patients treated with LLDs, 224 (27.7%) were in treatment with the high intensity lipid-lowering therapy. In particular, 149 (18.4%) patients used the high intensity LLDs and were adherent (MPR ≥ 80%), while 75 (9.3%) used the high-intensity lipid-lowering therapy but they were not adherent (MPR < 80%). In addition, 324 (40.1%) and 260 (32.2%) subjects were adherent and not adherent to low or moderate intensity lipid-lowering therapy, respectively. The high intensity strategy was adopted in 40 (4.9%) patients treated with LLDs, and 26 (65.0%) of them were adherent to the treatment.

The likelihood of LLDs prescription was increased in the patients affected by dyslipidemia, ischemic heart disease, and atherosclerosis. In addition, the patients with at least one specialist counseling and using a number of different molecules were more likely to be treated with LLDs. Conversely, the probability decreased in the patients affected by chronic respiratory diseases, heart failure, and with more comorbidities (Table 2).

**Table 2:** Probability of being treated with lipid-lowering drugs (LLDs) in the patients with diabetes.

	**Crude OR [95% CI]**	***P*-value**	**Adjusted OR [95% CI]**	***P*-value**
Gender, (M)	1.052 (0.848–1.304)	0.647	0.912 (0.704–1.183)	0.488
Age (years), median (IQR)	0.991 (0.982–1.000)	0.060	0.996 (0.985–1.008)	0.517
Lifestyle				
BMI	0.950 (0.925–0.976)	<0.001		
Alcohol abuse	1.389 (0.777–2.482)	0.268		
Smoking	0.807 (0.538–1.211)	0.301		
Comorbidity				
Dyslipidemia	3.218 (2.544–4.071)	<0.001	4.622 (3.444–6.204)	<0.001
Hypertension	1.296 (0.927–1.811)	0.129		
Ischemic heart disease	1.387 (1.091–1.763)	0.008	1.733 (1.296–2.318)	<0.001
Heart failure	0.689 (0.502–0.948)	0.022	0.657 (0.438–0.987)	0.043
Cerebrovascular disease	1.151 (0.926–1.430)	0.206		
Atherosclerosis	1.565 (1.216–2.014)	0.001	1.880 (1.381–2.558)	<0.001
Nephropathy	0.930 (0.750–1.154)	0.510		
CKD	1.006 (0.762–1.328)	0.967		
Obesity	0.835 (0.625–1.115)	0.221		
Neoplasm	0.975 (0.721–1.319)	0.871		
Psychic sphere disorders	0.810 (0.653–1.005)	0.055		
Arthritis and arthrosis	1.055 (0.850–1.308)	0.629		
Osteoporosis	1.097 (0.872–1.381)	0.429		
Chronic respiratory diseases	0.744 (0.599–0.924)	0.007	0.647 (0.486–0.861)	0.003
Gout and Metabolism disorders	1.105 (0.812–1.505)	0.525		
N. disease	1.049 (1.005–1.096)	0.027	0.876 (0.816–0.941)	<0.001
CCI	0.994 (0.939–1.051)	0.822		
Different molecules	1.074 (1.060–1.088)	<0.001	1.076 (1.060–1.094)	<0.001
Specialist counselling	3.405 (2.716–4.268)	<0.001	2.541 (1.970–3.279)	<0.001

*BMI, Body mass index; CCI, Charlson Comorbidity Index; CI, confidence interval; CKD, chronic kidney disease; OR, odds ratio; IQR, interquartile range; M, male*.

The LDL-C values below the therapeutic target resulted in 218 (18.8%) patients with high/very-high CVr; specifically, in 24.0 and 10.3% of the patients treated with or without LLDs (*p* < 0.01), respectively. The LDL-C target was reached by the 34.6% of patients prescribed with high intensity LLDs and adherent to the treatment.

The patients most likely to achieve the LDL-C target were men, older, and those with more comorbidities. Furthermore, the treatment with LLDs, especially the adherence to high intensity treatment, and specialist consulting were independent predictive factors for achieving the LDL-C target (Table 3).

**Table 3:** Predictive factors for reaching the low-density lipoprotein cholesterol (LDL-C) target in the patients with diabetes and at high and very-high CVr.

	**Crude OR [95% CI]**	***P*-value**	**Adjusted OR [95% CI]**	***P*-value**
Sex (M)	1.633 (1.210–2.203)	0.001	1.690 (1.231–2.322)	0.001
Age (years), median (IQR)	1.016 (1.003–1.030)	0.018	1.027 (1.011–1.044)	0.001
N. disease	1.087 (1.025–1.153)	0.006		
CCI	1.102 (1.034–1.175)	0.003	1.078 (1.007–1.154)	0.030
No treatment	–			
Low intensity no adherent	1.679 (1.051–2.680)	0.030	1.610 (0.994–2.607)	0.053
Low intensity adherent	2.956 (1.974–4.425)	<0.001	2.822 (1.848–4.309)	<0.001
High intensity no adherent	2.780 (1.440–5.638)	0.002	2.525 (1.284–4.965)	0.007
High intensity adherent	4.612 (2.868–7.417)	<0.001	4.123 (2.517–6.755)	<0.001
Different molecules	1.005 (1.003–1.007)	<0.001	1.009 (0.992–1.026)	0.305
Specialist counselling	1.993 (1.460–2.719)	0.001	1.726 (1.234–2.413)	0.001

*CCI, Charlson Comorbidity Index; CI, confidence interval; CV, cardiovascular risk; IQR, inter quartile range; M, male; MPR, medication possession ratio; OR: odds ratio*.

### Focus on Glucose-Lowering Drug Use

**Figure 2:**
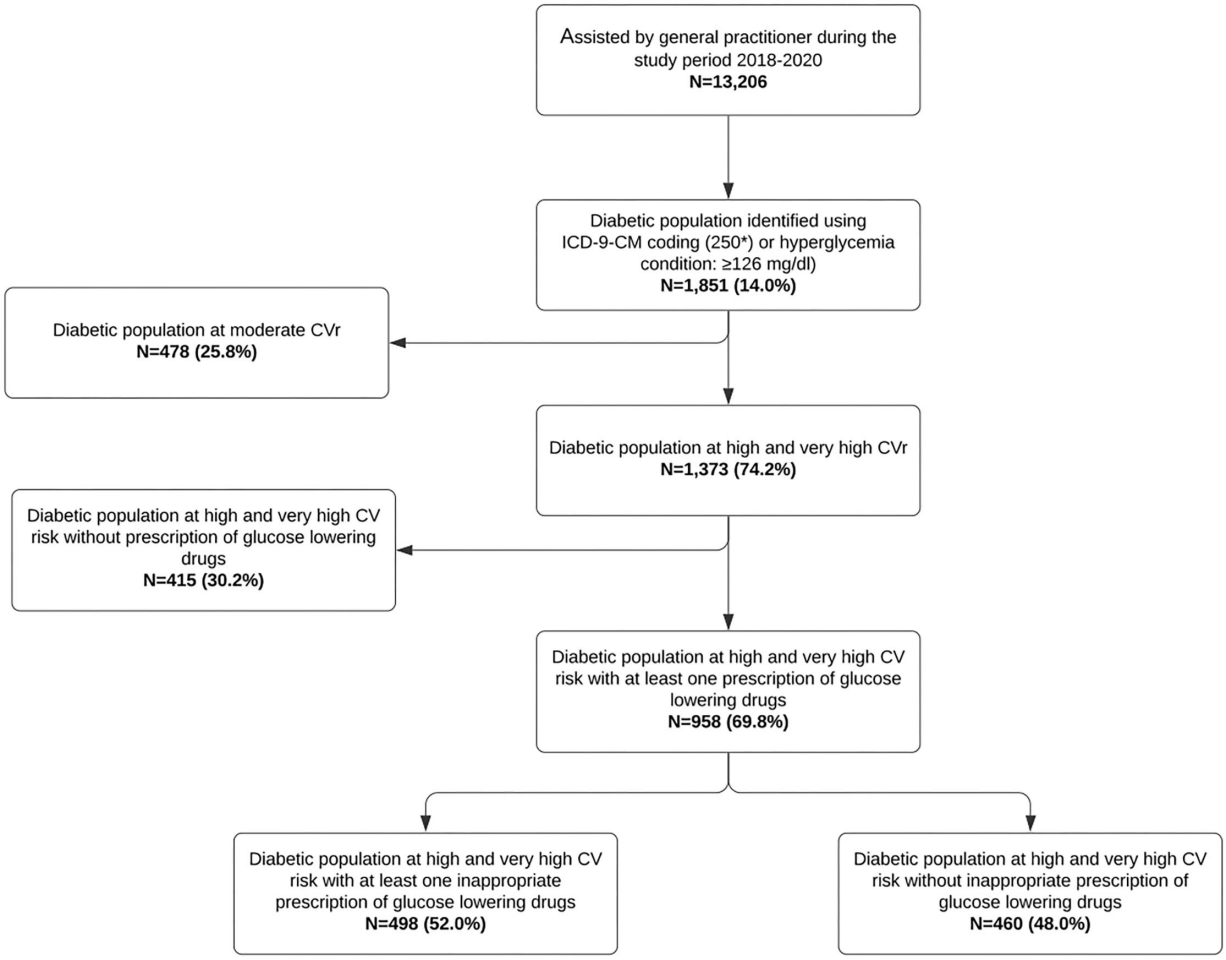
Identification of the study population: focus on diabetic profile.

Out of 1,373 patients at high/very-high CVr, 958 (69.8%) had at least one prescription of glucose-lowering drugs. The 52.0% (*n* = 498) of these therapies were not prescribed in agreement with the current guidelines to reduce the CVr (Figure 2). More specifically, 392 (40.9%) patients started the treatment or were treated only with metformin, while the remaining 106 (11.1%) were treated with metformin together with hypoglycemic agents other than GLP1-RA or SGLT2 inhibitors. Moreover, 30.4% of patients only treated with metformin, show HbA1c values >7%.

The predictors of appropriate treatment with glucose-lowering drugs were the following characteristics: older age, ischemic heart disease, heart failure, chronic kidney disease (CKD), and specialist counseling (Table 4).

**Table 4:** Predictors of the inappropriateness of hypoglycemic agents.

	**Crude OR [95% CI]**	***P*-value**	**Adjusted OR [95% CI]**	***P*-value**
Gender, (M)	0.938 (0.728–1.209)	0.619	1.014 (0.748–1.376)	0.926
Age (years), median (IQR)	0.972 (0.960–0.984)	<0.001	0.979 (0.965–0.993)	0.004
Lifestyle				
BMI	1.008 (0.976–1.041)	0.642		
Alcohol abuse	1.450 (0.735–2.860)	0.284		
Smoking	0.959 (0.607–1.516)	0.858		
Comorbidity				
Dyslipidemia	1.111 (0.844–1.461)	0.454		
Hypertension	1.237 (0.831–1.842)	0.294		
Ischemic heart disease	0.563 (0.424–0.747)	<0.001	0.676 (0.490–0.931)	0.017
Heart failure	0.353 (0.231–0.540)	<0.001	0.591 (0.362–0.965)	0.035
Cerebrovascular disease	0.793 (0.613–1.025)	0.077		
Atherosclerosis	1.085 (0.815–1.443)	0.576		
Nephropathy	0.755 (0.586–0.974)	0.031	1.011 (0.742–1.378)	0.944
CKD	0.202 (0.138–0.295)	<0.001	0.266 (0.178–0.396)	<0.001
Obesity	1.471 (1.032–2.096)	0.033	1.219 (0.805–1.847)	0.349
Neoplasm	0.700 (0.490–1.000)	0.050		
Psychic sphere disorders	0.815 (0.632–1.051)	0.115		
Arthritis and arthrosis	1.237 (0.959–1.594)	0.101		
Osteoporosis	0.920 (0.698–1.213)	0.554		
Chronic respiratory diseases	0.848 (0.654–1.100)	0.214		
Gout and metabolism disorders	0.787 (0.552–1.121)	0.184		
CCI	0.792 (0.735–0.853)	<0.001	0.950 (0.869–1.039)	0.258
LDL-C target (<70 mg/dL)	0.783 (0.564–1.088)	0.146		
High intensity lipid-lowering treatment and MPR ≥ 80%	0.697 (0.480–1.012)	0.058		
Different molecules	0.981 (0.968–0.995)	0.008	1.012 (0.994–1.030)	0.190
At least one registration of LDL-C	0.787 (0.539–1.149)	0.214		
Specialist counselling	0.655 (0.499–0.860)	0.002	0.692 (0.505–0.947)	0.022

*CCI, Charlson Comorbidity Index; CI, confidence interval; CKD, chronic kidney disease; IQR, inter quartile range; M, male; MPR, medication possession ratio; OR, odds ratio; LDL-C, low-density lipoprotein cholesterol*.

## Discussion

Diabetes represents a challenge, since it is one of the major risk factors for CVD, but also for its multiple associated comorbidities, which can involve micro- and macro-vasculature (16). However, the treatment of hyperglycemia alone has been effective in the patients with diabetes to prevent or delay the microvascular complications, but not for CVD (17). Reducing CVr in the patients with diabetes requires a multifactorial approach and optimal management of all risk factors. The randomized controlled trials demonstrated that careful treatment of hyperglycemia, multifactorial management of risk factors, and new treatment agents, such as SGLT2 inhibitors and GLP1-RA, significantly improve the cardiovascular outcomes (18). Therefore, this approach is strongly suggested in the last guidelines (5).

A wide discrepancy has been observed between the potential benefits seen in CV outcomes trials and the real-world care that patients affected by diabetes currently receive. In this study, the prescribing behavior of GPs in preventing the detrimental effects of DM on heart and vasculature was evaluated in a setting of patients with diabetes with high CVr.

In particular, careful assessment of the complete metabolic profile is of paramount importance to prevent the CV events in the patients with diabetes, independently of a single disease. The patients with high/very-high CVr have been monitored more carefully than the patients with moderate CVr, since the GPs have collected more frequent information on lifestyle and laboratory testing. These findings, in accordance with those previously reported (19), confirm the key role of GPs on the CVD risk assessment, management, and prevention. In fact, the careful assessment of metabolic profile by GPs results the most effective evidence-based approach to guide the decision-making process on starting treatment, and to ensure the right support that patients with DM need to reduce their CVr (20). Dyslipidemia is one of the five modifiable risk factors implicated in the increased risk of CV events (21); LDL-C is today acknowledged as a causal factor for atherosclerotic CVD (22). However, almost half of the patients with DM at high/very-high CVr have never tested for at least one LDL-C evaluation during the last 2 years. Monitoring must be implemented in these patients, with the goal to achieve the entirety of screened patients. In addition, it is important to highlight the need of periodically repeating the laboratory tests to have a systematic follow-up of the lipid profile trend to verify the treatment efficacy. The need to improve the correct monitoring of these patients is pressing since, during the study period, a great proportion of subjects remain without any LLD prescription, even if out of LDL-C target. Indeed, just above 50% of the patients were treated with LLDs, particularly when they were diagnosed for dyslipidemia, and about 90% of non-treated patients reported LDL-C values above the target. Moreover, almost 10% of the patients were either without any lipid-lowering treatment or with no LDL-C evaluation, suggesting a greater focus on the single disease, such as dyslipidemia, and careless management of the global CVr. The CV absolute risk assessment, using international suggested tools to estimate the 5- or 10-year CVr of an individual (23), could be really useful whether its importance is understood by the GPs and then, accurately transferred to the patients, but in primary prevention and/or in absence of the target organ involvement. Providing support to the GPs or programs helping the patients to better understand their actual risk, could have potential benefits on the prevention behaviors.

The LDL-C values on target resulted in a very small proportion of patients at high/very-high CVr (<20%) and, specifically in 24% of the treated patients. However, <20% of patients treated with LLDs used high intensity drugs and were adherent to the treatment, while <5% of patients adopted the high intensity strategy, although with the higher adherence compared with the overall study cohort. Underuse and discontinuation of medications for chronic diseases are common in the clinical practice, as showed in the previous studies (24). The lower adherence rates are mainly related to the perceived lack of therapeutic benefit, although the high adherence to therapy results in a nearly 3-fold greater probability of reaching the LDL-C therapeutic target, as defined by the EAS/ESC guidelines.

Nevertheless, most of the highly adherent cohort also failed to achieve the adequate LDL-C reduction. In spite of high adherence to the therapy, failure to achieve the recommended LDL-C levels might be attributable to the use of moderate doses and/or low to standard efficacy drugs (25). In our study, more than 70% of the treated patients used low-moderate efficacy drugs, while the patients prescribed with high intensity LLDs and adherent to the treatment showed about four times the probability of reaching the LDL-C therapeutic target, as reported in the previous studies. Nevertheless, the lack of optimal management of patients, suggested by our findings, agrees with the results of different observational studies that reported the poor control rates of LDL-C in the clinical settings (26–28).

In addition, a greater probability to achieve the LDL-C target was observed in male patients and in the elderly, as already known (29).

Several evidence highlight the need for collaborative approaches between the GPs and clinical specialists to improve the management of CVr factors (30). Furthermore, the more accurate follow-up of patients related to specialist counseling results in both better patients' compliance and treatment adherence (31). Accordingly, we found that the probability to be treated with LLDs and to achieve the LDL-C target increase more than 2.5-fold and almost 2-fold respectively, when the patients are referred to the specialist by GPs. Moreover, specialist counseling improves the management of high/very-high CVr in the patients with diabetes also reducing the inadequate prescriptions of glucose-lowering drugs. Nevertheless, we observed that most patients never underwent the specialist counseling. This evidence could be partially due to the too long waiting lists for the specialist consultations and the lack of a preferential way useful to improve the interaction between the GPs and specialists (31).

According to new evidence provided on CV safety in T2DM patients, the use of both GLP1-RA and SGLT2 inhibitors is strongly recommended in the patients with T2DM with high or very-high CVr, regardless of the metabolic compensation (5, 11).

Most of the patients with diabetes included in the study were prescribed with metformin alone or metformin with hypoglycemic drugs other than the SGLT2 inhibitors or GLP1-RA, even after the recommendation of guidelines. Moreover, almost one/third of the patients only treated with metformin, resulted above the HbA1c target. This inadequate choice by the GPs could be partially explained by their inability to independently prescribe SGLT2 inhibitors or GLP1-RA; indeed, these drugs can be prescribed by GPs, in Italy, only after a specific approval from the specialist. Therefore, more than half of the patients, never undergoing a specialist examination, could not start the treatment with the recommended drugs, GLP1-RA or SGLT2 inhibitors. The latest guidelines strongly recommend the use of SGLT2 inhibitors and GLP1-RAs that include the more recent and increasing evidence on CV protection in high/very-high risk in the patients with T2DM; however, these are not yet so extensively applied by all the physicians. Probably, more time is needed to notice the actual improvement in the patient management. Again, a most collaborative approach between the GPs and clinical specialists is surely needed to improve the management of high CVr patients with diabetes.

The probability to be in treatment with inappropriate glucose-lowering drugs was significantly lower in the patients affected by CKD. Indeed, the use of metformin should be avoided in patients with severe CKD, especially because of the increased risk of lactic acidosis (12, 32). Probably, other glucose-lowering drugs were preferred when the patients with diabetes are also affected by the CKD, regardless of the assessment of CVr.

From our findings, the patients with DM seem to be treated with low consideration for the potential CVr, while managing the diabetes condition cannot disregard the lipidic profile. Considering the individual risk profile of the patient with diabetes is an absolute requirement for the patient care. Our results suggest the urgent need in the real life to improve the management and treatment of the patients with diabetes at high/very-high CVr to reduce the burden of this disease on the health system.

### Strengths and Limitations

This is the first study observing the management of CVr in general clinical practice, carried out in a large cohort of patients with diabetes over a long-term study period, where both the lipid profile management and appropriate use of glucose-lowering drugs were evaluated according to CVr. However, we know that these data are the result of the voluntary collaboration of 10 GPs of a restricted area and may not be extended to the whole population of Italy. Nevertheless, the quality of information has been already shown in the previous drug-utilization studies.

We focused our investigation on the lipid profile modifying strategies and high CV risk assessment of the patients with diabetes in clinical practice, to verify whether lipid profile and CV risk are addressed in the patients with diabetes even independently of a diagnosis of dyslipidemia. Moreover, with the same purpose, the appropriate choice of glucose-lowering agents has been considered in the light of the reduction of CVr only, regardless of the metabolic target achievement. Therefore, the management of diabetes as a whole cannot be taken into account. However, median blood pressure and HbA1c values have been reported, to better characterize the patients.

We took into account all the traditional CVr factors, and CVr was considered in accordance with the EAS/ESC guidelines. We did not evaluate the Systematic Coronary Risk Estimation (SCORE) because all the variables needed for the SCORE calculation were not recorded for each selected patient. Consistently, the patients with high and very-high CVr could be slightly underestimated.

Additionally, the diagnoses might not be absolutely accurate, even if identified by ICD9 code, because they were recorded manually by the GPs into digital medical records.

In addition, all the information was gathered from the medical records of GPs and all data come from the clinical practice. Furthermore, the laboratory test results could be analyzed from different—certified—laboratory testing centers. However, the tests considered in this survey are not affected by a wide variability.

No information regarding the source of the prescription is recorded in the medical records. For this reason, it is not possible to know whether the prescriptions were performed directly by the GPs or suggested by the specialist. In Italy, however, all the treatments are registered in the medical records. Indeed, the drugs recommended by specialists are prescribed by GPs—free of charge for the citizen—and obviously GP can decide whether to prescribe the suggested drug or not.

## Conclusions

High/very-high CVr condition is poorly diagnosed and managed by GPs in Italy; the excessive GPs workload could play a role in this process.

In fact, the GPs in Italy should provide medical assistance to a great number of subjects, and the number of complex patients could be too heavy to be managed always in an optimal way; this crucial aspect can lead to the need to properly stratify CVr especially in the patients with diabetes, where atheromatosis, urinary albumin excretion, retinal disease, or worsening of renal function move the patients among the risk levels. Moreover, the patients properly allocated in the right risk level—high/very-high CVr—may not receive the proper pharmacologic treatment with LLDs, and just a residual percentage was prescribed with a high-intensity lipid-lowering strategy—achieving the lipidic goal with a significantly higher rate. Furthermore, we observed a non-ideal glucose-lowering treatment management in patiens affected by diabetes with high/very-high CVr compared to what recommended -and considered appropriate - by the current ADA and ESC guidelines.

Information on the innovative drugs and more effective therapeutic strategies should be conveyed to improve the patient management, especially when consistent guidelines are available. Last, a closer collaboration with medical specialists and clinical pharmacologist, not only in the advanced and often complicated stages of the disease, might be effective in implementing the preventive measures aimed to slow the disease progression and to improve the drug management in high/very-high CVr patients with diabetes.

## Data Availability Statement

The dataset generated for this study will not be made publicly available. Further inquires can be directed to the corresponding author.

## Author Contributions

VA, FS, SM, and RS: conceptualization. MR, CN, and EES: data curation. MAB and MR: formal analysis. MR, GSc, MAB, and VA: methodology. VA: project administration. FS, GM, and VA: supervision. GSq, EI, and VA: validation. MR, GSc, and GP: writing—original draft preparation. NI, GM, FS, and VA: writing—review and editing. All authors have read and agreed to the published version of the manuscript.

## Conflict of Interest

The authors declare that the research was conducted in the absence of any commercial or financial relationships that could be construed as a potential conflict of interest.

## Publisher's Note

All claims expressed in this article are solely those of the authors and do not necessarily represent those of their affiliated organizations, or those of the publisher, the editors and the reviewers. Any product that may be evaluated in this article, or claim that may be made by its manufacturer, is not guaranteed or endorsed by the publisher.
