# Supplementary material for: Management of High Cardiovascular Risk in Diabetic Patients: Focus on Low Density Lipoprotein Cholesterol and Appropriate Drug Use in General Practice
Source: Front Cardiovasc Med. 2021 Oct 25;8:749686. doi: 10.3389/fcvm.2021.749686 (PMC8572969; doi:10.3389/fcvm.2021.749686)
Supplement: Supplementary file 1 [file Table_1.docx]

**Supplementary Material**

**Table S1. Criteria for identifying diabetic patients at high/very high CVr**

| Atherosclerosis (ICD9=440*, 444*); |
| --- |
| Ischemic heart disease (ICD9=410* - 414*); |
| Cerebrovascular disease (ICD9=430* - 438) |
| Familial dyslipidemia (ICD9=272* and description: “familial dyslipidemia”) |
| Diabetes plus complications (ICD9=250.4-250.9) |
| Early onset diabetes of long duration (≥ 20 years) |
| Three or more major risk factors^a^ |
| Diabetes duration ≥ 10 years without plus any other additional risk factor^a^ |
| LDL-C ≥190 mg/dL |
| Total cholesterol ≥310 mg/dL |

**^a^** Age >50 years, chronic kidney disease, dyslipidemia, hypertension, obesity, smoking

Abbreviation: *CVr*, Cardiovascular Risk; *ICD9*, International Classification of Diseases, 9th revision; *LDL-C*, low density lipoprotein cholesterol.

**Table S2. Number of antihypertensive agents per patient used in hypertensive diabetics**

| Number of Drug Class | | High/very high CVr | | Moderate CVr | | Total patients | |
| --- | --- | --- | --- | --- | --- | --- | --- |
| 0 | | 180 (14.8) | | 35 (17.2) | | 215 (15.1) | |
| 1 | | 584(48.0) | | 108 (52.9) | | 692 (48.7) | |
| 2 | | 338 (27.8) | | 47 (23.0) | | 385 (27.1) | |
| >=3 | | 115 (9.4) | | 14 (6.9) | | 129 (9.1) | |
| Total | | **1217 (%)** | | **204 (%)** | | **1421 (%)** | |

Abbreviation: CVr: Cardiovascular Risk

**Table S3. Prevalence of use of antihypertensive agents in hypertensive diabetics**

| ATC-Drug Class | High/very high CVr  N. 1217 (%) | Moderate CVr  N. 204 (%) | Total patients  N. 1421 (%) |
| --- | --- | --- | --- |
| C02-[Antihypertensives](https://www.whocc.no/atc_ddd_index/?code=C02&showdescription=no) | 52 (4.3) | 9 (4.4) | 61 (4.3) |
| C03-[Diuretics](https://www.whocc.no/atc_ddd_index/?code=C03&showdescription=no) | 208 (17.1) | 24 (11.8) | 232 (16.3) |
| C07-Beta Blocking Agents | 397 (32.6) | 57 (27.9) | 454 (31.9) |
| C08-Calcium Channel Blockers | 198 (16.3) | 27 (13.2) | 225 (15.8) |
| C09-Agents Acting on the Renin-Angiotensin System | 770 (63.3) | 133 (65.2) | 903 (63.5) |

Abbreviation: *CVr*, Cardiovascular Risk

**Table S4. Number of glucose-lowering drugs per patient used in diabetics**

| Number of Drug Class | | High/very high CVr | | Moderate CVr | | Total patients | |
| --- | --- | --- | --- | --- | --- | --- | --- |
| 0 | | 415 (30.2) | | 173 (36.2) | | 588 (31.8) | |
| 1 | | 742 (54.0) | | 254 (53.1) | | 996 (53.8) | |
| 2 | | 186 (13.5) | | 46 (9.6) | | 232 (12.5) | |
| ≥3 | | 30 (2.2) | | 5 (1.0) | | 35 (1.9) | |
| Total | | **1373 (%)** | | **478 (%)** | | **1851 (%)** | |

Abbreviation: *CVr*, Cardiovascular Risk

**Table S5. Prevalence of use of glucose-lowering drugs in diabetics**

| ATC – Drug Class | High/very high CVr  N. 1373(%) | Moderate CVr  N. 478(%) | Total patients  N. 1851(%) |
| --- | --- | --- | --- |
| A10A*-Insulin | 242 (17.6) | 56 (11.7) | 298 (16.1) |
| A10BA-Biguanides | 561 (40.9) | 199 (41.6) | 760 (41.1) |
| A10BB-Sulfonylureas | 93(6.8) | 32 (6.7) | 125 (6.8) |
| A10BD-Combination of oral blood glucose-lowering drugs | 71 (5.2) | 25 (5.2) | 96 (5.2) |
| A10BF- Alpha glucosidase inhibitors | 63 (4.6) | 5 (1.0) | 68 (3.7) |
| A10BG-Thiazolidinediones | 14 (1.0) | 4 (0.8) | 18 (1.0) |
| A10BH-DPP4 inhibitors | 38 (2.8) | 8 (1.7) | 46 (2.5) |
| A10BJ-GLP1 analogues | 40 (2.9) | 11 (2.3) | 51 (2.8) |
| A10BK-SGLT2 inhibitors | 15 (1.1) | 4 (0.8) | 19 (1.0) |
| A10BX-Other blood glucose-lowering drugs, excl. insulins | 121 (8.8) | 25 (5.2) | 146 (7.9) |

Abbreviation: *CVr*, Cardiovascular Risk
